# Dr. Karnes’ Case

**Published:** 1895-08

**Authors:** W. W. Walker

**Affiliations:** Schulenburg, Texas


### Dr. Karnes' Case.

SCHULENBURG, TEXAS, July 27, 1895.

*Editor Texas Medical Journal:*

DEAR DOCTOR:—At the request of Dr. Karnes I write to say, Dr. Thompson, Professor of Surgery Medical Department Texas University, assisted by myself, Dr. Shropshire, of San Antonio, administering chloroform, operated on Dr. Karnes yesterday morning, cutting down upon and dissecting out the axillary nerves; the musculo-spinal nerve was not cut into as we expected; but the bullet had either passed through or cut a section out of it. A chip of bone had been carried from the scapula and left impinging upon the musculo-spinal nerve, and was inclosed in a mass of cicatricial tissue which was removed; about one inch of the nerve was about half an inch thick. This thick portion was slit longitudinally, the tissues brought in apposition with deep catgut and superficial silk sutures; a small iodoform gauze drain was placed in lower angle of wound. The result will be doubtful. Drs. Hadra, Hicks, Dupree, Young and Wortham were present. Dr. Thompson will probably give you a more extended and minute description of the operation.

Respectfully yours,

W. W. WALKER.

It will be remembered that Dr. Karnes was accidentally shot by his companion while out turkey hunting last winter; the ball of a Winchester entering the left shoulder. The wound healed readily, but left the arm paralyzed and very painful, especially the hand. The operation was for the purpose, if possible, of relieving this. Dr. Karnes is assistant superintendent S. W. Texas Insane Asylum at San Antonio. We are glad to learn he is doing well, with prospect of complete recovery.

